# Association Between Sleep and Motoric Cognitive Risk Syndrome Among Community-Dwelling Older Adults: Results From the China Health and Retirement Longitudinal Study

**DOI:** 10.3389/fnagi.2021.774167

**Published:** 2021-11-05

**Authors:** Weihao Xu, Anying Bai, Xin Huang, Yinghui Gao, Lin Liu

**Affiliations:** ^1^Haikou Cadre’s sanitarium of Hainan Military Region, Haikou, China; ^2^Department of Epidemiology and Biostatistics, School of Population Medicine and Public Health, Chinese Academy of Medical Sciences and Peking Union Medical College, Beijing, China; ^3^Department of Geriatric Cardiology, The Second Medical Center & National Clinical Research Center for Geriatric Diseases, Chinese PLA General Hospital, Beijing, China; ^4^PKU-UPenn Sleep Center, Peking University International Hospital, Beijing, China; ^5^Department of Pulmonary and Critical Care Medicine, The Second Medical Center & National Clinical Research Center for Geriatric Diseases, Chinese PLA General Hospital, Beijing, China

**Keywords:** sleep duration, sleep quality, motoric cognitive risk syndrome, community, older adults

## Abstract

**Background**: Sleep is increasingly recognized as an important lifestyle contributor to health; however, its relationship with Motoric cognitive risk syndrome (MCR) is still unclear. The present study aimed to examine the associations between sleep duration, sleep quality, and MCR among community-dwelling Chinese older adults.

**Methods**: We recruited 5,387 participants aged ≥60 years from the China Health and Retirement Longitudinal Study (CHARLS). Sleep-related variables including night sleep duration and sleep quality were assessed *via* self-reported questionnaires. MCR syndrome was defined as cognitive complaints and slow gait speed without dementia or impaired mobility. Multivariate logistic regression analysis was performed to explore the associations between sleep-related variables and MCR after controlling for all potential confounders including demographic characteristics, lifestyle factors, and comorbidities.

**Results**: We found that sleep duration was significantly associated with MCR, and the multivariate-adjusted odds ratios (OR) were highest for those with the shortest (<6 h *OR* = 1.55, 95% *CI* = 1.18–2.04) and longest (≥10 h *OR* = 1.73, 95% *CI* = 1.03–2.91) sleep durations. Moreover, an increasing frequency of self-perceived poor sleep quality was significantly associated with MCR in the adjusted model (3–4 days *OR* = 1.58, 95% *CI* = 1.16–2.17; 5–7 days *OR* = 1.81, 95% *CI* = 1.37–2.40).

**Conclusions**: Our study indicated an inverted U-shaped association between night sleep duration and MCR. Poor sleep quality was also associated with higher odds of MCR in community-dwelling Chinese elders. Longitudinal studies with a larger population size are needed to establish causality in the future and further explore potential action mechanisms.

## Introduction

In recent decades, the proportion of people aged 60 years or older has increased rapidly and is projected to be greater than one-fifth of the global population in 2050 (Prince et al., 2013). Consequently, the burden of dementia and cognitive impairment is also increasing. China has the largest population of patients with dementia in the world (Jia et al., 2020) and is expected to host almost half of the global dementia population by 2050 (Chhetri et al., 2020). Motoric cognitive risk (MCR) syndrome is a predementia syndrome characterized by subjective cognitive complaints and objective slow gait in older individuals without dementia or any mobility disability (Verghese et al., 2013). It has recently been suggested that providers use MCR syndrome diagnosis as an indicator to screen for adverse health outcomes (Chhetri et al., 2017) including falls (Callisaya et al., 2016), disability (Doi et al., 2017), dementia (Doi et al., 2017), cardiovascular disease (Meiner et al., 2020), and mortality (Beauchet et al., 2019). A clearer understanding of potentially modifiable determinants of MCR, enabling effective preventative intervention design, would help alleviate the increasing individual and societal burdens of dementia.

Sleep is a complex and crucial physiological process for the brain; it can restore the brain’s functional capacity, maintain homeostasis, and is necessary for survival. Older adults tend to experience less nighttime sleep and have more sleep disturbances compared to younger adults (Ohayon et al., 2004). As previous experimental research demonstrated, a decrease in sleep quality and quantity might elevate the concentration of β-amyloid, a hallmark of Alzheimer’s disease (AD; Xie et al., 2013), and therefore lead to a higher risk of dementia and different types of cognitive impairment. Numerous studies have investigated the various associations between sleep duration and cognition, but the results are conflicting. Some cohort studies observed sleep duration to be statistically significantly associated with cognitive decline and incident dementia (Virta et al., 2013; Devore et al., 2014; Bokenberger et al., 2017; Ohara et al., 2018), whereas others did not reach this conclusion (Tworoger et al., 2006; Benito-León et al., 2009; Yaffe et al., 2011; Keage et al., 2012; Potvin et al., 2012; Blackwell et al., 2014). Some reported an inverted U-shaped association between sleep duration and the risk of dementia (Chen et al., 2016; Bubu et al., 2017) or cognitive decline (Xu et al., 2011), which means that, compared to intermediate sleep duration, short or long sleep durations are associated with cognitive decline or dementia. However, still, others found that longer sleep duration was associated with poorer cognitive function (Benito-León et al., 2009; Faubel et al., 2009). This discrepancy may be due to various evaluations of sleep duration and cognitive function, different characteristics among the studied cohorts, and differences in study length. Along with sleep duration, the relationship between sleep quality and cognitive performance has also been studied, and mixed findings have been reported. Some studies showed that poorer sleep quality measured by the Pittsburgh Sleep Quality Index (PSQI) was associated with weaker working memory (Noort et al., 2016) and executive function (Nebes et al., 2009), while others failed to find an association between subjective sleep quality and cognitive performance (Stepanski et al., 1989; Miyata et al., 2013). However, to the best of our knowledge, there is currently little evidence supporting the U-shaped association between sleep duration and MCR in developing countries, especially in China. Even fewer have investigated the relationship between sleep quality and MCR among Chinese older adults.

Sleep-related factors are potentially modifiable. Early identification and control of sleep-related predictors of MCR might have important clinical implications and generate early avenues for the prevention of progression to dementia, specifically in the large Chinese elderly population. Therefore, this study used data from the China Health and Retirement Longitudinal Study (CHARLS), a nationally representative longitudinal study, to examine the inverted U-shaped association between sleep duration and probability of MCR, and to investigate the associations between sleep quality and MCR among community-based Chinese older adults.

## Materials and Methods

### Study Participants

We used the national baseline data from the CHARLS to investigate the association between sleep duration, sleep quality, and MCR among Chinese middle-aged and older adults adjusting for socio-demographical factors, lifestyle factors, and comorbidities. The CHARLS baseline data collection took place from June 2011 to March 2012 and included 17,708 respondents from 10,257 households. The target population was middle-aged and older adults and their spouses aged 45 years and above and currently residing in a household in China (Sonnega et al., 2014). Ethical approval for data collection on human subjects was received from Peking University’s institutional review board (IRB). The CHARLS data are publicly available on the China Health and Retirement Longitudinal Study website^1^.

### Definition of MCR

MCR syndrome has been defined as cognitive complaints and slow gait speed without dementia or impaired mobility. In our study, cognitive complaints were assessed using a self-reported questionnaire about memory loss: “How would you rate your memory at the present time?” Respondents were asked to rank their answers on a scale of five values: “excellent”, “very good”, “good”, “fair”, and “poor”. Those who reported “fair” or “poor” were recorded as having cognitive complaints. Gait speed was computed by the average time respondents took to walk along a straight 2.5 m path twice and was used to assess physical performance. The cutoff slow gait speed was 1.0 standard deviations or below age- (65–69, 70–74, ≥75 years old) and sex- (male and female) appropriate mean values of gait speed in our cohort (Verghese et al., 2014).

### Sleep-Related Variables

Total nighttime sleep duration data was obtained from the question “During the past month, how many hours of actual sleep did you get at night (average hours for one night)?” and respondents were categorized into five groups according to their answers based on previous research (Yokoyama et al., 2010; Arora et al., 2011; Paudel et al., 2013): <6 h, 6 to <7 h, 7 to <9 h, 9 to <10 h, and ≥10 h.

Sleep quality was assessed by the subjects’ responses regarding the validity of the following statement: “My sleep was restless.” They were classified into four groups based on their answers: “1 Rarely or none of the time (<1 day),” “2 Some or a little of the time (1–2 days),” “3 Occasionally or a moderate amount of the time (3–4 days), ” and “4 Most or all of the time (5–7 days).”

### Covariates

Demographic characteristics, lifestyle factors, and self-reported comorbidities were controlled as covariates in this study. Demographic characteristics included age, gender, marital status, and educational level. Lifestyle factors included body mass index (BMI; kg/m^2^), smoking history, and alcohol consumption. Health comorbidities included a self-reported history of hypertension, diabetes, chronic lung disease, kidney disease, and cardiac disease.

### Statistical Analysis

All variables are presented as the mean ± standard deviation, or frequencies and percentages. Baseline characteristics between participants with different nighttime sleep durations were compared using unpaired *t* or *Χ*^2^ tests.

Multivariate logistic regression analysis was performed to study the association between sleep-related variables and MCR while considering age, gender, marital status, educational level, smoking history, alcohol consumption, BMI, hypertension, diabetes, chronic lung disease, kidney disease, and cardiac disease as potential confounders. Results were reported as adjusted odds ratios and the 95% confidence interval (CI).

All reported p values were two-tailed with a significance level of 0.05. All analyses were performed using STATA software (version 14.0; Stata Corp LP. TX).

## Results

### Characteristics of Participants According to Sleep Duration

**Figure 1:**
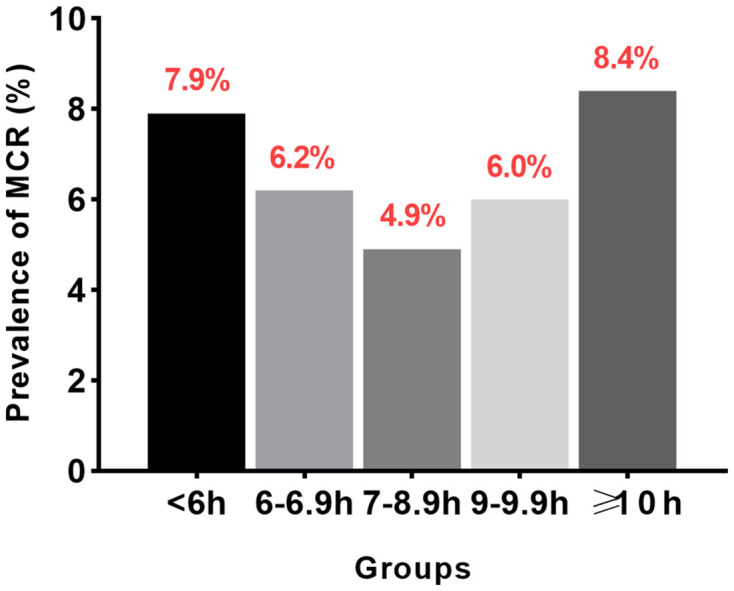
Prevalence of motoric cognitive risk (MCR) in different sleep duration groups.

Table 1 summarizes the baseline characteristics of the studied participants. There were 1,871 (34.7%), 1,069 (19.8%), 2,006 (37.2%), 216 (4.0%), and 225 (4.18%) participants who reported nightly sleeping <6 h, 6–6.9 h, 7–8.9 h, 9–9.9 h, and ≥10 h, respectively. The average age was older in the longest and shortest sleep duration groups, and the shortest sleep duration group had a lower proportion of males than the other sleep duration groups. Moreover, we observed a U-shaped prevalence of MCR between groups, with the longest and shortest sleep duration groups being the most prevalent (Figure 1). There were significant associations between sleep duration and age, gender, marital status, educational level, BMI, smoking history, history of diabetes, cardiac disease, kidney disease, and chronic lung diseases vs. the odds of MCR. There were no significant associations between sleep duration and alcohol consumption or history of hypertension.

**Table 1:** Characteristics of study participants and comparing between groups (*n* = 5,387).

	<6 h (*n* = 1,871)	6–6.9 h (*n* = 1,069)	7–8.9 h (*n* = 2,006)	9–9.9 h (*n* = 216)	≥10 h (*n* = 225)	*P* value
Age, years (mean ± *SD*)	68.2 ± 6.7	67.3 ± 6.2	67.2 ± 6.2	68.3 ± 6.5	69.9 ± 7.0	<0.001
Male, *n* (%)	827 (44.2)	585 (54.7)	1,079 (53.8)	115 (53.2)	118 (52.4)	<0.001
Married, *n* (%)	1,423 (76.1)	882 (82.5)	1,647 (82.1)	175 (81.0)	155 (68.9)	<0.001
Education level, *n* (%)						<0.001
≤ Primary school	1,633 (87.3)	800 (74.8)	1,611 (80.3)	187 (86.6)	208 (92.4)
Middle school	184 (9.8)	174 (16.3)	249 (12.4)	20 (9.3)	15 (6.7)
≥ High school	54 (2.9)	95 (8.9)	145 (7.2)	9 (4.2)	2 (0.9)	
BMI, (mean ± SD)	22.5 ± 3.8	23.1 ± 3.9	23.2 ± 3.8	22.6 ± 3.7	22.7 ± 3.7	<0.001
Current Smokers, *n*(%)	540 (28.9)	331 (31.0)	678 (33.8)	74 (34.3)	71 (31.6)	<0.018
Current drinkers, *n*(%)	544 (29.1)	340 (31.8)	641 (32.0)	61 (28.2)	81 (36.0)	<0.099
History of hypertension, *n* (%)	589 (31.5)	300 (28.1)	606 (30.2)	77 (35.6)	72 (32.0)	<0.144
History of diabetes, *n* (%)	127 (6.8)	68 (6.4)	148 (7.4)	6 (2.8)	8 (3.6)	<0.031
History of cardiac disease, *n* (%)	291 (15.6)	180 (16.8)	283 (14.1)	17 (7.9)	34 (15.1)	<0.010
History of kidney disease, *n* (%)	146 (7.8)	64 (6.0)	107 (5.3)	9 (4.2)	13 (5.8)	<0.015
History of Chronic Lung Diseases, *n* (%)	310 (16.6)	141 (13.2)	246 (12.3)	17 (7.9)	27 (12.0)	<0.001
MCR, *n* (%)	147 (7.9)	66 (6.2)	98 (4.9)	13 (6.0)	19 (8.4)	<0.003

*SD, standard deviation; BMI, body mass index; MCR, motoric cognitive risk syndrome*.

### Association Between Sleep Duration and MCR

Table 2 demonstrates the association between sleep duration and odds of MCR in logistic regression analyses. We used a sleep duration of 7–8.9 h/night as the reference category. Compared to the median night sleep duration, we found that a shorter nighttime sleep duration (<6 h) was significantly associated with 1.55 times higher odds of MCR (95% CI 1.18–2.04), and a longer nighttime sleep duration (≥10 h) was significantly associated with a 1.73 times higher chance of MCR diagnosis (95% CI 1.03–2.91) after adjusting for all potential covariables. No significant relationship between 6–6.9 h/night or 9–9.9 h/night and MCR was found.

**Table 2:** Associations between sleep duration and MCR.

	Model 1^a^	Model 2^b^	Model 3^c^
<6 h	1.66 (1.28–2.16)	1.60 (1.23–2.09)	1.55 (1.18–2.04)
6–6.9 h	1.28 (0.93–1.77)	1.29 (0.93–1.78)	1.37 (0.99–1.91)
7–8.9 h	1.00	1.00	1.00
9–9.9 h	1.25 (0.69–2.26)	1.25 (0.69–2.27)	1.12 (0.64–2.21)
≥10 h	1.80 (1.08–3.00)	1.81 (1.08–3.02)	1.73 (1.03–2.91)

*MCR, motoric cognitive risk syndrome. a, Model 1 was a crude model; b, Model 2 adjusted for age and gender; c, Model 3 adjusted for age, gender, marital status, educational level, smoking status, drinking status, body mass index, hypertension, diabetes, chronic lung disease, kidney disease, and cardiac disease*.

### Association Between Sleep Quality and MCR

**Figure 2:**
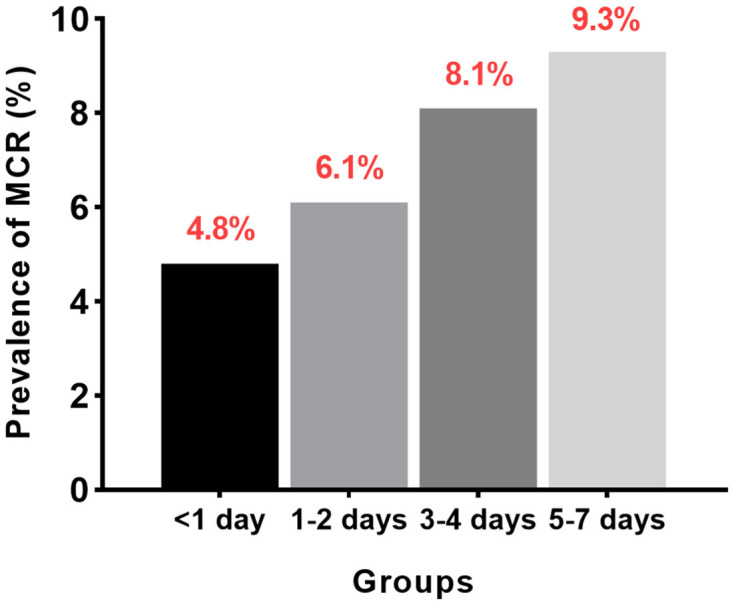
Prevalence of MCR in different sleep quality groups.

Table 3 presents the relationship between sleep quality and odds of MCR in the adjusted and unadjusted models. An increasing frequency of self-perceived poor sleep quality was significantly associated with MCR in both the crude and adjusted models (Figure 2). Compared to participants who rarely felt restless after sleep, those who reported feeling restless after sleep most or all of the time were significantly associated with 1.81 times higher odds of MCR (95% CI 1.37–2.40) after adjusting for age, gender, marital status, educational level, smoking status, alcohol use, BMI, hypertension, diabetes, chronic lung disease, kidney disease, and cardiac disease.

**Table 3:** Associations between sleep quality and MCR.

	Model 1^a^	Model 2^b^	Model 3^c^
Rarely or none of the time	1.00	1.00	1.00
(<1 day)		
Some or a little of the time	1.30 (0.93–1.81)	1.26 (0.90–1.75)	1.23 (0.88–1.72)
(1–2 days)	
Occasionally or a moderate	1.76 (1.29–2.39)	1.64 (1.20–2.23)	1.58 (1.16–2.17)
amount of the time		
(3–4 days)		
Most or all of the time	2.06 (1.57–2.69)	1.93 (1.47–2.53)	1.81 (1.37–2.40)
(5–7 days)

*MCR, motoric cognitive risk syndrome. a, Model 1 was a crude model; b, Model 2 adjusted for age and gender; c, Model 3 adjusted for age, gender, marital status, educational level, smoking status, drinking status, body mass index, hypertension, diabetes, chronic lung disease, kidney disease, and cardiac disease*.

## Discussion

In the nationally representative cohort of community-dwelling Chinese older adults, we found that, compared to median night sleep duration, shorter and longer night sleep durations were significantly associated with increased odds of MCR diagnosis, indicating an inverted U-shaped association between nighttime sleep duration and cognitive performance. Moreover, an increasing frequency in self-reported poor sleep quality was also significantly associated with higher odds of MCR after adjusting for potential confounders, including demographic characteristics, lifestyle factors, and self-reported comorbidities.

Several epidemiological studies on Asian populations have also reported a U-shaped association between sleep duration and cognitive function, which is in concordance with our results. Ohara et al. (2018) investigated the association between self-reported daily sleep duration and risk of dementia by grouping all participants into five categories (<5.0, 5.0–6.9, 7.0–7.9, 8.0–9.9, ≥10.0 h/night) in a Japanese elderly cohort. They observed a U-shaped association between sleep duration and dementia during the 10-year follow-up period. Xu et al. (2011) found that short (3–4 h/day) or long sleep durations (≥10 h) were an important factor independently associated with memory impairment among 28 67 + -year-old community-dwelling adults in Guangzhou. A prospective study among 7,444 community-dwelling women (aged 65–80) showed that both short-duration (≤6 h/night) and long-duration (≥8 h/night) sleepers had a greater cognitive decline and an increased risk of cognitive impairments, including dementia, compared to those with self-reported sleep of 7 h/night during an average of more than 7 years of follow-up. Another study among older Chinese women identified a significant U-shaped association between a higher dementia risk with either short (≤6 h/night) or long (≥8 h/night) sleep durations (vs. 7 h/night; Chen et al., 2016). In contrast, Benito-León et al. (2009) observed a significant positive association between long self-reported sleep duration (≥9 h) and incident dementia. For short-duration sleepers (≤5 h), after controlling for relevant confounders, the relationship was attenuated and no longer significant (Benito-León et al., 2009; Kawada, 2015).

Previous studies have also investigated the relationship between changes in sleep duration and cognitive decline, but the results were contradictory.

Hahn et al. (2014) used data from a population-based sample of 214 Swedish adults aged 75 and over and found that reduced sleep was associated with a 75% increase in all-cause dementia risk at a 3-year follow-up. However, Westwood et al. (2017) reported that transitioning to sleeping >9 h over a mean period of 13 years was associated with an increased risk of all-cause dementia. Moreover, Bokenberger et al. (2017) demonstrated that short and extended time in bed (TIB) among older adults predicted increased dementia incidence in the 17 years following the initial sleep analysis. The variation in follow-up period, outcome measurements, cohort size, and studied population might partially explain the discrepancies between results.

Although several studies have examined the relationship between sleep duration and cognitive function, few have investigated the relationship between sleep duration and odds of MCR. Only one cross-sectional study among 940 Chinese older adults aged ≥65 years in the Ningbo Community Study on Aging (NCSA) reported that longer night sleep duration (>8.5 h) was associated with higher odds of MCR compared to a shorter night sleep duration (<8 h). The relatively few participants with extremely short sleep durations prevented this study from exploring a possible U-shaped association (Zeng et al., 2021).

Besides sleep duration, we also found that subjective poor sleep quality was significantly associated with higher odds of MCR after adjusting for confounders. Few previous studies have assessed whether subjective sleep disturbance predicts cognitive decline or cognitive impairment among older adults, and the results were inconsistent. Tworoger et al. (2006) found that women aged 70–81 years who regularly had difficulty falling or staying asleep scored 0.11 units lower on the global cognition score (95% CI: −0.22, 0.01) compared to those who rarely had difficulty sleeping. Jelicic et al. (2002) reported a negative relationship between sleep complaints and cognitive performance at a follow-up among 838 middle-aged and older adults (≥50 years). Cricco et al. (2001) observed that self-reported symptoms of insomnia independently predicted the incidence of cognitive decline 2 years later in older men, but not older women. Inconsistent conclusions across the current literature about the relationship between sleep quality and cognitive function among older adults could be explained by the variability of the subjective sleep quality indicators used. More objective methods and tools to measure sleep disturbance (such as wrist actigraphy) among larger samples of older adults might be needed to clarify these associations in the future.

The mechanisms underlying the association between sleep disturbance and odds of MCR remain unclear, although several potential biological pathways between sleep disturbance and cognitive function have been identified. Firstly, reduced or prolonged sleep duration has been found to be related to changes in the brain’s ventricular volume (Lo et al., 2014) and total cerebral brain volume (Westwood et al., 2017) as well as cortical thickness (Spira et al., 2016), all of which influence cognitive performance. Secondly, neuroinflammation caused by interleukin 6 and C-reactive protein activation might occur after sleep deprivation (Atienza et al., 2018), which might mediate age-related cognitive impairment (Legdeur et al., 2018). Thirdly, poor sleep quality may derail metabolic and endocrine function, which could induce cognitive impairments. For instance, chronic sleep deprivation is linked to an increase in amyloid-β plaque formation, which is a hallmark of AD (Kang et al., 2009). Besides AD, poor sleep quality may exacerbate or accelerate the neurodegenerative process of different types of dementia.

Our study has several strengths. First, it is the first study to use a nationally representative cohort of community-dwelling Chinese adults to evaluate the association between sleep disturbance and MCR, which adds generalizability to our findings. Moreover, our results revealed that self-reported sleep duration and subjective poor sleep quality may be useful clinical tools to help predict persons at risk of progressing to dementia. Second, respondents were asked about sleep duration without being given example durations, and the classification of different nighttime sleep periods was set based on previous literature, both of which contributed to the identification of a reliable U-shaped association between sleep duration and odds of MCR. Third, a wide range of potential confounding parameters including demographic background, lifestyle factors, and history of comorbidities was adjusted for within the multivariable analyses, thus providing robust statistical power. However, several limitations should also be acknowledged. Firstly, the assessments of nighttime sleep duration were collected based on self-reporting in this study, which may have been influenced by recall bias. Although self-reported sleep has been shown to reflect objective assessments of sleep accurately (Biddle et al., 2015), more objective measurements are required in future studies. Secondly, this investigation was an observational study, so no causal relationships could be demonstrated. Lastly, the sleep quality of participants was not fully investigated. More detailed measurements, like the PSQI, a self-rated questionnaire that assesses sleep quality and disturbances over a 1-month time interval (Buysse et al., 1989), would be needed to comprehensively evaluate the sleep quality of all participants.

## Conclusion

In summary, this study demonstrated that shorter or longer sleep durations than average and subjective poor sleep quality were associated with increased odds of MCR. These findings have substantial public health implications and emphasize the need to improve the quality and quantity of sleep to prevent the prevalence of pre-dementia symptoms in developing countries where the number of older adults with dementia is expected to increase. In the future, longitudinal studies with comprehensive assessments of sleep disturbance among a larger population are required to establish causality and further explore potential mechanisms between sleep-related factors and MCR.

## Data Availability Statement

The original contributions presented in the study are included in the article, further inquiries can be directed to the corresponding author/s.

## Ethics Statement

The studies involving human participants were reviewed and approved by Peking University’s Institutional Review Board. The patients/participants provided their written informed consent to participate in this study.

## Author Contributions

WX wrote and participated in all aspects of this research, including the field investigation. AB and XH analyzed the data and interpreted the results. YG and LL designed this research. All authors reviewed the manuscript and agree to be accountable for the content of the work. All authors contributed to the article and approved the submitted version.

## Conflict of Interest

The authors declare that the research was conducted in the absence of any commercial or financial relationships that could be construed as a potential conflict of interest.

## Publisher’s Note

All claims expressed in this article are solely those of the authors and do not necessarily represent those of their affiliated organizations, or those of the publisher, the editors and the reviewers. Any product that may be evaluated in this article, or claim that may be made by its manufacturer, is not guaranteed or endorsed by the publisher.

## Footnotes

^1^ http://charls.pku.edu.cn/
